# Effect of Farming System and Season on Proximate Composition, Fatty Acid Profile, Antioxidant Activity, and Physicochemical Properties of Retail Cow Milk

**DOI:** 10.3390/ani13233637

**Published:** 2023-11-24

**Authors:** Eleni Kasapidou, Roxani-Aikaterini Stergioudi, Vasileios Papadopoulos, Paraskevi Mitlianga, Georgios Papatzimos, Maria-Anastasia Karatzia, Michail Amanatidis, Vasiliki Tortoka, Ekaterini Tsiftsi, Antonia Aggou, Zoitsa Basdagianni

**Affiliations:** 1 Department of Agriculture, University of Western Macedonia, Terma Kontopoulou, 53100 Florina, Greece; vpapadopoulos@uowm.gr (V.P.); papatzimos@hotmail.com (G.P.);; 2 Department of Chemical Engineering, University of Western Macedonia, Koila, 50100 Kozani, Greecepmitliagka@uowm.gr (P.M.);; 3 Research Institute of Animal Science, HAO-Demeter, 58100 Giannitsa, Greece; karatzia@rias.gr; 4 School of Agriculture, Department of Animal Production, Aristotle University of Thessaloniki, 54124 Thessaloniki, Greecebasdagianni@agro.auth.gr (Z.B.)

**Keywords:** cow milk, retail, conventional, organic, season, proximate composition, fatty acid composition, antioxidant profile, physicochemical characteristics

## Abstract

**Simple Summary:**

Consumer perception of organic cow milk is associated with the assumption that organic milk differs from conventionally produced milk. This study examined the effect of the farming system and season on the quality attributes of retail cow milk. Over one year, milk samples originating from conventional and organic production systems were collected monthly and assessed for their nutritional content, proximate composition, fatty acid profile, nutritional value, total phenolic content, antioxidant activity, and physicochemical characteristics. Results show that nutrient content remained consistent across production systems, except for a slightly higher fat level in organic milk. Fatty acid composition varied between systems and seasons, with organically produced milk showing higher polyunsaturated fatty acids. Nutritional indices related to lipid quality showed no significant differences between production systems and seasons. Total phenolic content remained consistent, while antioxidant activity was system-dependent and seasonal. Season influenced both free radical-scavenging activity (DPPH) and ferric reducing-antioxidant power (FRAP). Milk physicochemical characteristics, including pH, electrical conductivity, and freezing-point depression, were mainly influenced by season. Overall, this study provides detailed insights into the multifaceted interactions between production systems, seasons, and diverse characteristics of retail cow milk.

**Abstract:**

Consumers differentiate milk-quality characteristics in relation to the production system, but data on retail milk composition are limited. This study investigated how farming methods and seasons affect proximate composition, fatty acid profile, antioxidant activity, and physicochemical characteristics of commercial cow’s milk. Milk samples, both conventional (*n* = 84, 7 manufacturers) and organic (*n* = 24, 2 manufacturers), were collected monthly over a year. Farming system did not significantly affect milk composition other than fat content, whereas seasonal effects were notable, impacting the contents of ash, protein, and added water. Fatty acid composition exhibited variations influenced by both production system and season. Compared to conventionally produced milk, organically produced milk exhibited higher levels of polyunsaturated fatty acids (4.54 vs. 3.88, *p* < 0.01) and a lower atherogenicity index (2.23 vs. 2.50, *p* < 0.05). The antioxidant activity showed that conventionally produced milk exhibited better radical-scavenging activity (DPPH) (14.54 vs. 12.30 μM TE/mL, *p* < 0.01). Seasonal variations were evident in both free radical-scavenging activity (DPPH), with values of 12.29 μM TE/mL in winter and 15.58 μM TE/mL in spring (*p* < 0.05), and ferric reducing-antioxidant power (FRAP), with levels of 21.81 μM TE/mL in autumn and 27.94 μM TE/mL in spring (*p* < 0.05). Season significantly affected (*p* < 0.001) milk pH, electrical conductivity, refractive index, and freezing-point depression. In conclusion, this study showed that the farming system has a limited impact on retail milk quality compared to the significant influence of season.

## 1. Introduction

Cow milk is an essential source of macro- and micro-nutrients and is vital in the human diet [1,2]. Previous research has shown that parameters such as diet, breed, genotype, farming system, and season affect the nutritional quality of cow milk [2,3,4,5]. From a consumer perspective, the production system is a crucial factor influencing milk quality. In this context, organic milk is perceived as kinder to the environment, animals, and people, produced without antibiotics, added hormones, synthetic chemicals, and genetic modification, potentially offering advantages for human health [6]. Additionally, there is a growing awareness among consumers regarding the nutritional value of milk. In this regard, particular components of milk, notably antioxidants, are garnering interest due to their crucial role in sustaining the balance between pro-oxidant and antioxidant processes within the human body. Finally, analyses of the physicochemical attributes, including pH, electrical conductivity, refractive index, and freezing-point depression, serve as valuable and efficient indicators of milk-quality characteristics, and are typically carried out within the industry.

However, the existing data on the composition of cow milk primarily stem from studies conducted on farm samples. Additionally, Butler et al. [4] expressed concerns about the applicability of research findings in assessing the quality of milk available to consumers due to (a) the potential lack of representativeness of individual farms selected for sampling, concerning the broader production system within a country, and (b) the potential impact of processing conditions throughout the supply chain on milk composition.

Furthermore, in their daily lives, consumers are faced with decisions regarding the type of milk they include in their diets, with choices typically encompassing organic, free-range, and conventional milk in food retail outlets. However, there is limited research on the composition of retail cow milk. Regarding the antioxidant activity of milk and dairy products that has been extensively studied [7], there are limited studies on the antioxidant profile of cow milk at retail level [8,9]. Similarly, studies on the physicochemical properties of bovine milk available to consumers are also scarce.

Information on the quality of cow milk at the retail level is scarce. Butler et al. [4] and Vicini et al. [10] conducted studies investigating the influence of farming systems on milk proximate composition and identified that the production system impacted macronutrient composition, specifically fat and protein content. Stergiadis et al. [11] and Butler et al. [4] reported seasonal variations in fatty acid composition in organically produced milk. In the context of Greece, as far as we are aware, there are no studies on the effect of the production system and season on the quality of retail cow milk. In Greece, produced milk is categorized as conventional (96% of production) or organic (4% of production), as shown in Table 1. There has been a growing trend toward the intensification of dairy farms in Greece, leading to increased utilization of concentrated feed. Additionally, the prevailing climatic conditions, characterized by low annual rainfall and dry summers, contribute to limited pasture production throughout the year [12]. This limitation on forage intake contrasts with the conditions in other European countries. However, there are no studies on the diets of dairy cows reared in organic and conventional farms in Greece. Nevertheless, organic milk production is subject to European legislation for the production of organic products [13].

Thus, the study aimed to investigate the effect of production systems (conventional and organic) and season (spring, summer, autumn and winter) on proximate composition, fatty acid composition, antioxidant profile, and physicochemical characteristics of retail cow milk produced in Greece.

## 2. Materials and Methods

### 2.1. Sampling

Milk samples (*n* = 108) from 9 brands were collected every month, from November 2019 to October 2020 from four major supermarket retailers located in the city of Florina, Greece (40°46′55″ N 21°24′35″ E). All samples were full-fat homogenized milk and were either conventionally (*n* = 7 brands) or organically produced (*n* = 2 brands). Milk samples were pasteurized under either high-temperature–short-time (HTST) or ultra-high-temperature (UHT) conditions. For extended-shelf-life samples, a combination of pasteurization and microfiltration was applied. Care was taken to purchase the milk samples on the day following its production at the dairy plant, ensuring that analyses for all samples commenced on day 1 post-production.

The selected samples had to meet the following criteria (a) to be widely available in food stores and (b) to bare the “Greek Produce” mark, which certifies the origin of goods and services produced in Greece. The “Greek Produce” mark is an official trademark of the Greek State, and it is optional. The application of the mark on agricultural and livestock products requires that production, rearing, and harvest take place within Greek territory, whereas for processed products, it is necessary that the basic raw materials originate from Greece. Furthermore, care was taken to avoid purchasing private-label milk from the same producer as the branded milk. All cow milk samples meeting the specified criteria and that were available in supermarket retailers were included in the study, while considering that Greece has 40% self-sufficiency in cow’s milk [12].

Food stores (supermarkets) were located within a 6 km radius from the laboratory where samples were stored and analyzed. Following purchase, milk samples were transported to the laboratory within 1 h. On arrival at the laboratory, milk samples were thoroughly mixed and decanted into 15 mL Falcon tubes. Specimens intended for analyzing the proximate composition, and determination of the physicochemical characteristics were kept at 4 °C and analyzed within 24 h of collection. Specimens destined for the determination of fatty acid composition and antioxidant profile were kept at −20 °C until analyzed. The declared milk composition of each milk sample was noted.

### 2.2. Milk Proximate Composition

Milk proximate composition (fat, protein, lactose, and solids-non-fat content) was determined with a milk analyzer (Lactostar, Funke Gerber, Berlin, Germany). The moisture, ash, and total solids were calculated using these equations:

Moisture (%) = 100 − Total Solids (%)(1)

Ash (%) = Solids non Fat (%) − (Protein (%) + Lactose (%))(2)

Total Solids (%) = Solids non Fat (%) + Fat (%)(3)

The percentage of added water was determined according to the following equation:

(4) $$Added\;water=\frac{\left(-0.525-{FPD}_{x}\right)\times 100}{-0.525}$$

where FPD_x_ is the freezing-point depression of the sample. In the case of a negative value being found, it was treated as a zero.

Milk with a freezing point of −0.525 °C or lower may be presumed to be water free [15].

### 2.3. Determination of Fatty Acid Profile

Milk samples were thawed overnight at 4 °C, and the following day the milk lipids were extracted with a chloroform/methanol solution (1:2 *v*/*v*) according to the method of Bligh and Dyer [16]. Solvents contained 0.01% (*wt*/*v*) of t-butyl-hydroxytoluene (BHT) to prevent oxidation of the unsaturated fatty acids during extraction. Fatty acid methyl esters were prepared from the extracted lipids using base-catalyzed methanolysis of the glycerides using KOH in methanol, according to the method ISO–IDF 15,884 [17] of the International Organization for Standardization. Fatty acid methyl ester analysis was performed on an Agilent Technologies 6890N GC (Agilent Technologies, Inc., Santa Clara, CA, USA) equipped with a flame ionization detector (FID) and a 60 m × 0.25 mm i.d., 0.25 μm film thickness DB-23 (50% Cyanopropyl 50% dimethyl polysiloxane) capillary column (Model Number: Agilent 122 2362). The injector temperature was set at 250 °C. The oven temperature was programmed from 110 °C (held for 6 min) to 165 °C at 1 °C/min (held for 13 min), to 195 °C at 15 °C/min (held for 22 min) and to 230 °C at 7 °C/min (hold for 7 min). The carrier gas was helium at 0.7 mL/min, and the injection volume was set at 3 μL, and the e split ratio was 1:50. The injection was performed using an Agilent 7683 Series auto-sampler (Sigma-Aldrich, Taufkirchen, Germany). Fatty acids were identified using three commercial-standard mixtures: (a) 37 component FAME mix (Supelco, 47885-U, Sigma-Aldrich, Taufkirchen, Germany) (b) PUFA-2, Animal source (Supelco, 47015-U, Sigma-Aldrich, Taufkirchen, Germany), and (c) a mixture of cis- and trans-9,11- and -10,12-octadecadienoic acid methyl esters (Sigma, O5632–250MG, Sigma-Aldrich, Taufkirchen, Germany) as reference standards. Fatty acids were quantified using peak-area measurement, and the results are expressed as percent (%) of the total peak areas for all quantified acids. Fatty acids were grouped as saturated fatty acids (SFA), monounsaturated fatty acids (MUFA), and polyunsaturated fatty acids (PUFA). Saturated fatty acids were also classified into short-, medium- and long-chained fatty acids as follows:

Short-chain saturated fatty acids (SCSFA) = C6:0 + C8:0 + C10:0 + C11:0

Medium-chain saturated fatty acids (MCSFA) = C12:0 + C13:0 + C14:0 + C15:0 + C16:0

Long-chain saturated fatty acids (MCSFA) = C17:0 + C18:0 + C20:0

#### Milk Fat Nutritional Indices

The nutritional value of the ingestible fat was evaluated through an examination of the fatty acid profile, involving the calculation of various indices. These specific indices are commonly utilized in dairy products, as indicated in the recent review by Chen and Liu [18], which consolidates research on fatty acid profiles published since 2000. The aim is to enhance our understanding of how the composition of fatty acids relates to human health.

Polyunsaturated fatty acid/Saturated fatty acid ratio:

PUFA/SFA = ∑PUFA/∑SFA

Atherogenicity index:

AI = [C12:0 + (4 × C14:0) + C16:0]/∑UFA

Thrombogenicity index:

TI = (C14:0 + C16:0 + C18:0)/[(0.5 × ∑MUFA) + (0.5 × ∑*n*-6PUFA) + (3 × ∑*n*-3PUFA) + (*n*-3/*n*-6)]

Hypocholesterolaemic: hypercholesterolaemic fatty acid ratio (h/H):

h/H = (C18:1*n*-9 cis + ∑PUFA)/(C12:0 + C14:0 + C16:0)

Health-promoting index:

HPI = ∑UFA/[C12:0 + (4 × C14:0) + C16:0]

The desaturation indices (Δ^9^ desaturase activity) were determined according to Hanuš et al. [19].

$$DI_{14}=\frac{C14:1\;cis9}{C14:0+C14:1\;cis9}\;\;\;DI_{16}=\frac{C16:1\;cis9}{C16:0+C16:1\;cis9}\;\;\;DI_{18}=\frac{C18:1\;cis9}{C18:0+C18:1\;cis9}$$

### 2.4. Total Phenolic Content and Antioxidant Activities

The total phenolic content (TPC) of the samples was determined using the Folin–Ciocalteu method [20]. The results are presented as milligrams of gallic acid equivalents (GAE) per ml of milk. Free radical-scavenging activity was assessed using the DPPH (2,2-diphenyl-1-picrylhydrazyl) method, following the protocol of Sanchez-Moreno et al. [21] with slight modifications. The results were expressed as μΜ of Trolox equivalents (TE) per ml of milk, where Trolox is a water-soluble analogue of vitamin E: 6-hydroxy-2,5,7,8-tetramethylchroman-2-carboxylic acid. The reducing power activity of the samples was measured using the FRAP (ferric reducing-antioxidant power) method, as reported by Pulido et al. [22], with minor changes. The results were expressed as μΜ of Trolox equivalents (TE) per ml of milk. Finally, the total antioxidant capacity of the milk samples was determined using the ABTS [2,2′-azinobis-(3-ethylbenzothiazoline-6-sulfonic acid)] method, following the procedure outlined by Re et al. [23] with slight adjustments. The results were expressed as μΜ of Trolox equivalents (TE) per ml of sample.

### 2.5. Milk Physicochemical Properties

Prior to conducting the analysis, the samples were placed in a temperature-controlled water bath to attain a room temperature of 20 °C. Subsequently, they were gently mixed by inverting the sample container several times, ensuring not to generate any frothing. The pH of the milk was assessed using a glass electrode containing an integrated temperature sensor (model 5014T, Crison Instruments, Barcelona, Spain) within a pH meter (model GLP 21, Crison Instruments, Barcelona, Spain). This pH meter was calibrated in accordance with the manufacturer’s guidelines using standard buffer solutions with pH values of 4.0 and 7.0. The electrical conductivity of the samples was determined utilizing a conductometer (model GLP 31, Crison Instruments, Barcelona, Spain) coupled with a sodium ion-selective electrode (model 50 70, Crison Instruments, Barcelona, Spain), calibrated with buffer solutions of 147 μS/cm, 1413 μS/cm, and 12.88 mS/cm. The refractive index and Brix value were measured using a digital refractometer that included a Peltier thermostat (model DR6000-T, Krüss, Hamburg, Germany). Finally, the freezing point depression (FPD) was assessed using a milk cryoscope (model 4250, Advanced Instruments Inc., Norwood, MA, USA). The cryoscope was calibrated using 422 (−408 m°C) and 621 (−600 m°C) reference solutions (3LA023 and 3LA033, Advanced Instruments Inc.), and the instrument performance was checked using Lactrol^®^ 530 reference solution (3LA030, Advanced Instruments Inc.). The measurement range of the cryoscope was 0–1000 m°C. 

### 2.6. Statistical Analysis

General linear models were used to investigate differences in milk proximate composition, fatty acid composition, antioxidant profile and physicochemical characteristics due to (a) farming system and (b) season. Farming system and season were fixed factors. Interactions between the main factors were also assessed, but the data are not presented as there was no significant interaction observed across all examined variables. Pearson correlation coefficients were determined for the relation between total phenolic content and antioxidant capacity, and for lactose content and freezing-point depression. The results are presented as least square means and standard error of difference (SED). SPSS software (version 28.0, SPSS Inc., Chicago, IL, USA) was used for data analysis. The results were considered to be significant when the *p*-values were <0.05.

## 3. Results and Discussion

### 3.1. Milk Characteristics According to Commercial Brands

The characteristics of the milk samples in relation to type, shelf life, and label, are provided in Table 2. Among the samples selected for this study, 77.78% were derived from the conventional production system, while the remaining 22.22% were sourced from the organic production system. Additionally, most of the samples (77.78%) carried brand labels, while private labels constituted only 22.22% of the total. Most of the samples had a shelf life of seven days.

The declared nutrient content of the milk displayed consistency between the two production systems (Table 3). The average fat, protein, and carbohydrate (sugar) content for both production systems was 3.62, 3.26, and 4.69 g/100 mL, respectively. The mean saturated-fatty-acid content was 2.31 g/100 mL. The principal carbohydrate present in milk is lactose, a disaccharide consisting of two simple sugars, glucose and galactose [24]. When comparing the two farming methods, organic milk displayed slightly higher fat and protein content, while its saturated-fatty-acid content was marginally lower than conventionally produced milk. 

### 3.2. Milk Proximate Composition

The proximate composition of milk is summarized in Table 4. In terms of protein and lactose contents, they closely align with the figures reported by the Hellenic Agricultural Organization-Demeter [25] for raw cow milk in 2019 and 2020 (protein: 3.31 g/100 g, lactose: 4.76 g/100 g for both years). However, it is noteworthy that the fat content of raw unprocessed milk, as per the Hellenic Agricultural Organization-Demeter [25], was 3.93 g/100 g in 2019 and 3.97 g/100 g in 2020. Milk gross composition was within the range reported in the scientific literature [26,27] and complied with Greek legislation governing milk available to consumers [28]. As per the Code of Foodstuffs, Beverages, and Objects of Common Use [28], the minimum required values for fat, protein, and solids-non-fat content are 3.5 g/100 g, 2.9 g/100 g, and 8.5 g/100 g, respectively. Furthermore, there were no deviations from the established tolerance [29] limits between declared and determined fat, protein, and lactose content.

Differences in milk composition between the farming system and seasons are shown in Table 4. Farming system did not affect milk composition with the exception of fat content that was higher in organically produced milk. However, since the fat content of milk is adjusted by the milk processors and organically produced milk samples had a higher declared fat content the observed difference cannot be related to the farming system. With regard to conventionally produced milk, it was found that Stergiadis et al. [30] reported lower fat, protein, and lactose contents in commercial milk in the United Kingdom, in comparison to the contents reported in the present study. Butler et al. [4] observed a significantly higher fat content in retail organic milk in northeast UK compared to conventionally produced milk, with no differences found in milk protein content. Vicini et al. [10] found that organically produced milk had increased protein content compared to conventionally produced milk and attributed this difference to farm management practices, such as reduced reliance on fat supplementation in cow diets or lower milk yield. Increased milk yield is generally associated with lower fat and protein contents due to a dilution effect. A highly significant (*p* < 0.001) season effect was observed in the contents of ash, protein and added water, whereas a significant effect (*p* < 0.05) was found in the contents of lactose and solids-non-fat. Ash content was lower in winter in relation to spring, summer and autumn. Chen et al. [31] found a seasonal effect on raw bulk-tank milk-ash content with the highest content observed in summer and the minimum content observed in autumn. On the contrary, Lindmark-Månsson et al. [32] reported a highly significant effect of season on ash content from raw milk collected from dairy plants and related it to factors such as breed and feed.

Protein content was higher in winter in comparison to the other seasons. The seasonal effect on milk protein has been reported by other workers as well. Lindmark-Månsson et al. [32] reported a higher protein content in milk collected in autumn in relation to milk collected in spring. Heck et al. [3] found higher protein levels in winter milk in relation to summer milk in pooled bulk-tank-milk samples. The latter workers related this effect to dietary changes, particularly to the higher concentrate-to-forage ratio in winter, which produces milk with a higher protein content.

Higher levels of added water were found in spring and summer in relation to autumn and summer. However, the added water content did not exceed the specified tolerance limit of 3% during milk collection. According to the study’s cryoscope instruction manual, this tolerance limit is calculated using Equation (4) with a base value of (FPD_x_) −0.540 °C, which results in 2.8% of added water. This value is rounded up to 3% to account for minor natural variations and measurement errors.

Extraneous water in milk is related to adulteration with water [33] but also due to unintentional water addition resulting from handling and processing procedures in dairy plants [34]. The higher content of extraneous water in spring and summer can be related to the reduced milk production due to changes in the proportions of concentrates in diet [35] and the higher temperatures [36] leading farmers to adulterate milk with water to increase the volume of milk. Nonetheless, given that the economic value of milk is determined by its fat and protein content, this approach is rarely employed, as it can lead to farmers being penalized by dairy companies.

In summary, the production system had a minimal impact on milk composition, except for the slightly higher fat content in organically produced milk, which can be attributed to standardization by the processors rather than the production system. Conversely, the season had a significant effect on milk components, including ash, protein, lactose, solids-non-fat, and added-water levels, reflecting the influence of dietary and environmental factors.

### 3.3. Milk Fatty Acid Profile and Nutritional Value

Milk fatty acid composition either as individual fatty acids or as different lipid classes are presented in Table 5. Capric (C10:0), lauric (C12:0), myristic (C14:0), palmitic (C16:0), and stearic (C18:0) acids were the predominant saturated fatty acids in milk samples from both production systems and seasons. Oleic acid (C18:1 *cis*-9) and linoleic acid (C18:2 *n*-6 *cis*) were the major monounsaturated and polyunsaturated fatty acids, respectively. The milk composition in both production systems and across all seasons resembles the composition reported in other studies [3,4,12,33,37] that analyzed milk from various European countries. Cows on the organic system produced milk with significantly lower levels (*p* < 0.01–0.05) of myristic (C14:0), myristoleic (C14:1), pentadecanoic (C15:0), and γ-linolenic (C18:3 *n*-6) acids. Organic milk had significantly higher levels (*p* < 0.01) of stearic (C18:0) and linoleic (C18:2 *n*-6 *cis*) acids. The impact of the season on fatty acid composition showed variability. Significant variations (*p* < 0.001–0.05) were observed in the concentrations of butyric (C4:0), capric (C10:0), lauric (C12:0), myristic (C14:0), pentadecanoic (C15:0), palmitoleic (C16:1), and linolenic (C18:3 *n*-3) acids. For the other fatty acids, the seasonal effect was found to be insignificant.

The effect of production system and season is in partial agreement with the data reported in other studies [3,4,11,37] examining retail or farm samples. Butler et al. [4] also reported higher levels of stearic and linoleic acids in organic retail milk in the UK compared to conventional milk. The same authors found that organic milk also had higher levels of myristic (C14:0) and pentadecanoic (C15:0). Tzamaloukas et al. [37] reported also that farming system affected the levels of fatty acids in a similar pattern to the one observed in the present study. With regard to season, higher levels of palmitoleic acid (C16:1) were reported in spring and summer [4,37], whereas other researchers found lower levels in summer in relation to autumn [3]. The effect of season in the even-chain SFA is not consistent with the results reported in the previous studies. The effect of the farming system and season was more prevalent in other research works [3,4,11,37], in comparison to the current study where both factors affected only a limited number of fatty acids. Moreover, the lack of consistency between the data from the present study and data from other studies, conducted in different countries, indicates mainly variations in the type of diets used in different production systems and seasons in other regions.

Finally, it is important to note that neither farming system nor season had a significant effect (*p* > 0.05) on the levels of the nutritionally important conjugated linoleic acid (CLA), as reported in other studies. Ruminant products such as milk, cheese, and meat are important sources of CLA in the human diet [38]. In general, forage intake can affect milk fatty acid composition, but it is dependent on parameters such as the type of forage, variation in pasture availability, and stage of grass growth maturity.

The effects of the farming system and season on milk lipid classes is presented in Table 5. The production system had a significant impact (*p* < 0.01–*p* < 0.05) on the levels of long-chain saturated fatty acids (LCSFA), polyunsaturated fatty acids (PUFA), odd-chain fatty acids (OCFA), and *n*-6 fatty acids. The levels of LCSFA and OCFA were significantly lower in milk samples from organic origin, whereas the levels of PUFA and *n*-6 fatty acids were higher in the same type of milk. The impact of the production system on milk lipid classes has been identified by other researchers. Butler et al. [4] reported significantly higher levels of PUFA in retail organically produced milk. A similar pattern to the one of the present was reported by Tzamaloukas et al. [37] that examined fatty acid composition in conventional and organic bulk-tank bovine milk. Season affected (*p* < 0.05) the levels of short-chain saturated fatty acids (SCSFA) mainly in summer and autumn. This is related to the seasonal changes of capric acid (C10:0), which is the major acid in this particular lipid class. Similar to this study, Frelich et al. [39] found higher levels of SCSFA in milk collected from November to April in comparison to milk collected from May to October and related this seasonal effect to variations in the feeding system such as changes from silage-based diets to pasture-based diets.

The endogenous synthesis of unsaturated fatty acids, certain monounsaturated fatty acids and almost all conjugated linoleic acid, is controlled by the Δ^9^-desaturase activity (Stearoyl-CoA desaturase) [40]. Neither production system nor season significantly impacted Δ^9^-desaturase activity (Table 5). The C14:1/C14:0 is considered a reliable indicator to assess the effect of dietary changes on the Δ^9^-desaturase activity since myristic acid (C14:0) in the milk is produced by de novo synthesis in the mammary gland [41]. Stergiadis et al. [11] reported lower Δ^9^-desaturase activity in retail organic produce in the UK in comparison to the conventionally produced one. With regard to the effect of season, Lock and Garnsworthy [41] found greater Δ^9^-desaturase activity in the summer, and, in combination to the presence of greater amounts of short-chain fatty acids, attributed this effect to changes in the pattern of fatty acids produced de novo in the mammary gland when animals are fed on fresh grass. Regardless, there is still limited understanding of the factors that influence changes in de novo fatty acid production within the mammary gland.

The impact of both the production system and season on nutritional indices related to lipid quality is detailed in Table 6. The production system affected (*p* < 0.05) the Atherogenicity Index (AI), where lower values were found in organically produced milk. Stergiadis et al. [11] also reported a lower but not significantly different AI in a similar study in organically produced milk. The same authors reported a highly significant monthly effect on milk AI, where higher values were found in winter months and lower values were found in summer months. With regard to the thombogenicity index (TI), neither the production system nor the season had any effect observed. In the previously reported study of Stergiadis et al. [11], a highly significant farming system and the month’s effect was observed. TI was lower in milk from the organic farms and similarly to our study, but the month’s effect did not follow the pattern observed in the present study.

AI illustrates the relation between saturated fatty acids (SFA), such as lauric (C12:0), myristic (C14:0), and palmitic acid (C16:0), which are considered to promote atherosclerosis, and unsaturated fatty acids (UFA), regarded as having anti-atherogenic properties because they inhibit plaque formation and reduce the levels of phospholipids, cholesterol, and esterified fatty acids. TI, assesses the clot-forming potential of fatty acids in blood vessels [42]. It is considered advantageous for human health to have low values for both indices, preferably less than 3 [43]. In our current study, the TI value exceeded the recommended threshold. The values of both AI and TI are within the reported range for either market or farm samples (AI: 1.96–4.07) and (TI: 2.16–3.88) [11,37,44].

Furthermore, both farming system and season had no effect (*p* > 0.05) on the hypocholesterolaemic: hypercholesterolaemic (h/H) ratio, which is employed to represent the relationship between the hypocholesterolemic and the hypercholesterolemic fatty acids with higher values being desirable. The mean h/H ratio was 0.59. Kiczorowska et al. [45] reported that extensive production systems such as traditional or organic resulted in higher h/H ratio values as compared to milk from intensively reared cows. The previous researchers reported lower h/H ratio values (0.37–0.43) than the ones found in our study.

Similarly, neither production system nor season affected (*p* > 0.05) the health-promoting index (HPI). Delgadillo-Puga et al. [46] reported higher HPI in organically produced milk than conventionally produced milk in temperate regions, and the reported range for HPI was 0.40–0.56 which was similar to the one observed in this study. Dairy products with a high HPI value are regarded as more beneficial for human health, as the HPI is the inverse of the thrombogenic index (TI) [18].

The PUFA/SFA ratio was not influenced by either the production system or the season. Barlowska et al. [47] reported a significant seasonal effect (summer vs. winter) in individual milk samples, where higher PUFA/SFA ratio values were found in the samples collected in the summer. The PUFA/SFA ratio values that have been reported fall within the range documented by Barlowska et al. [47], which is 0.05 to 0.07. PUFA/SFA ratio is widely used to evaluate the nutritional quality of ingested fats, indicating that polyunsaturated fatty acids (PUFA) may reduce low-density lipoprotein cholesterol and overall serum cholesterol levels, while saturated fatty acids (SFA) tend to elevate serum cholesterol levels. Thus, a higher PUFA/SFA ratio is linked to a more-favorable impact on cardiovascular health [18], with a recommended threshold value fixed at 0.45 [48]. Typically, ruminant milk has a low PUFA/SFA ratio, and as reported by Gibson et al. [48], who conducted a review of cohort studies, there is no consistent evidence linking the consumption of dairy products with a higher risk of cardiovascular disease.

In summary, neither the farming system nor season significantly impacted the fatty acid composition and milk-fat nutritional indices, except for the higher PUFA content and lower AI value in organically produced milk, highlighting that the production system is the primary factor influencing milk fatty acid composition.

### 3.4. Milk Total Phenolic Content and Antioxidant Activity

Table 7 shows the effect of the farming system and season on milk total phenolic content and antioxidant profile. The total phenolic content of milk was not affected by the production system and season, while it was lower than the reported values for heat-treated milk samples [9,49]. Kuhnen et al. [50] found that the amounts of phenolic compounds differed between samples from various production systems and seasons. However, the effect of diet on the phenolic content of milk has not been extensively studied.

With regard to the antioxidant activity, the production system had a significant effect (*p* < 0.01) on the free radical-scavenging activity (DPPH), whereas there was no effect on the ferric reducing-antioxidant power (FRAP) or in the total antioxidant capacity (ABTS). Conventionally produced milk had better radical-scavenging activity (DPPH) than organically produced milk. On the other hand, season had a significant impact (*p* < 0.05) on the free radical-scavenging activity (DPPH) and the ferric reducing-antioxidant power (FRAP) with higher levels observed in spring and summer. Kuhnen et al. [50] also observed greater ferric reducing-antioxidant power (FRAP) in summer than in winter. However, to the best of our knowledge, no other studies examine the effects of the production system and season on the antioxidant profile in retail cow milk, which would allow us to compare our findings.

The differences in the total phenolic content were not directly proportional to the antioxidant activity of the milk. This is because milk contains various components with antioxidant functions beyond polyphenols. Contributors to the antioxidant capacity of milk include sulfur-containing amino acids like cysteine, as well as components such as phosphate, vitamins A and E, carotenoids, zinc, selenium, and enzyme systems like superoxide dismutase, catalase, and glutathione peroxidase [51]. Pearson correlation coefficients demonstrated a positive relation between total phenolic content (TPC) and all three indices for antioxidant capacity. In detail, this applies for TPC and DPPH (r = 0.261 and *p* < 0.01), for TPC and FRAP (r = 0.232 and *p* < 0.01), and TPC and ABTS (r = 0.318 and *p* < 0.001). Cloetens et al. [52] reported that the total antioxidant capacity (ABTS) method was more useful in determining the antioxidant capacity in milk, whereas Becker et al. [53] reported that comprehending the antioxidant properties of food products necessitates the utilization of diverse methods.

Data comparison from this study to those in the existing literature is difficult due to variations in measurement units (Trolox equivalent antioxidant capacity or percentage of radical scavenging) and the use of different analytical methods. The fact that the antioxidant capacity was not expressed in the same patterns in samples from the same category is related to the different mechanisms for each assay. According to Cloetens et al. [52], due to the variety of techniques used in the determination of total antioxidant capacity in milk, a clear pattern is not evident despite observed differences between examined samples.

In summary, milk’s total phenolic content was unaffected by both production system and season. In terms of antioxidant activity, the production system significantly affected free radical scavenging (DPPH) in favor of conventionally produced milk, while season had a significant impact on both DPPH and ferric reducing-antioxidant power (FRAP), with higher levels during spring and summer.

### 3.5. Milk Physicochemical Characteristics

Differences in milk physicochemical characteristics due to the production system and season are shown in Table 8. A highly significant (*p* < 0.001) season effect was observed for milk pH. Chen et al. [31] also reported a similar seasonal variation in milk pH with higher values observed in the spring months. The latter researchers related pH differences to variations in protein and mineral content. Macheboeuf et al. [54] reported that turning animals to pasture produces changes in milk mineral content that results in an increase of 0.02 in milk pH. Milk pH was 6.74 within the acceptable range (6.6–6.8) for commercial milk [55]. Milk pH affects coagulation time, rate of firming into gel, and maximum firmness [56,57].

Production system and season significantly impacted electrical conductivity (*p* < 0.01 and *p* < 0.001, respectively). Cermanová et al. [58] also found lower electrical coductivity values in organically produced milk. According to Mabrouk and Petty [59], factors such as season and feed can affect milk conductivity in cow milk. The conductive properties of milk are associated with the presence of salts, primarily composed of chlorides, phosphates, citrates, carbonates, and bicarbonates of potassium, sodium, calcium, and magnesium whose concentrations can vary as they are influenced by parameters such as animal breed, season, feed, and stage of lactation. Fox et al. [15] reported that ions (particularly Na^+^, K^+^, and Cl^−^) are responsible for most of the electrical conductivity of milk, which is increased by the bacterial fermentation of lactose to lactic acid. Finally, Stergiadis et al. [30] have also documented changes in the relative concentrations of minerals in retail cow milk samples on an annual basis. Electrical conductivity values are within the range reported by Kailasapathy [60] (4–5 mS/cm) for cow milk, but are lower than the values reported in the study of Căpriţă et al. [61] for pasteurized milk (5.24–5.28 mS/cm).

A highly significant (*p* < 0.001) season effect was found in both values. The Brix value, and subsequently refractive index value, can be used as an indirect method to provide information on the total solid contents of milk, although the correlation between total solids content and Brix/refractive index is not exceptionally high [62]. Furthermore, despite the limitations of the method application of portable refractometers by small dairy producers, it is encouraged because it is a rapid, nondestructive, relative precise, which is an easy-to-use and cost-effective technique for monitoring the quality of milk. Moreover, the relationship between the refractive index of milk and its total solids content varies with changes in the concentration and composition of the solutes in milk [15]. The refractive index values of the examined samples were within the range (1.3440–1.3485) presented by Fox et al. [15]. The discrepancy between the absence of a seasonal effect on the total solids content (Table 4) and the highly significant seasonal impact on Brix and refractive index values (Table 8) may be linked to the scattering of light by casein micelles and fat globules, which could potentially affect the accuracy of the measurements [15]. In the present study, milk samples were homogeneous; thus, the effect of fat globules can be ruled out. On the other hand, Chen et al. [31] reported a significant correlation (*p* < 0.05) between the casein micelle size and total solids content. However, these researchers did not observe a seasonal variation in casein micelle size. In contrast, Holt and Muir [63] noted a pronounced seasonal trend in the average size of casein micelles, with smaller sizes during the summer than in winter. Nevertheless, the influence of season on indirect methods for assessing the total solids content in milk warrants further investigation. Nowadays, the use of portable refractometers offers a quick, non-invasive, precise, and budget-friendly method for farmers and small dairy producers to assess milk quality [62,64]. In this respect, the effect of the production system and season on milk’s Brix and refractive index values was studied.

The farming system did not affect (*p* > 0.05) the freezing-point depression of milk, whereas a highly significant (*p* < 0.001) season effect was observed. Higher freezing-point depressions were observed in spring and summer in relation to autumn and spring. Variations in freezing-point depression follow variations in extraneous (added) water content (Table 4). Changes in the freezing-point depression are not only associated with the added water but also are due to changes in diet and the environmental temperature. Bjerg et al. [65] reported that increased water intake might affect milk freezing-point depression in pasture-fed cows. Additionally, Henno et al. [66] found that season significantly affected freezing-point depression. Higher values were observed during the grazing period, and the authors associated them with increased water consumption caused by the increased environmental temperature. Freezing-point depression is primarily affected (≈80%) by the levels of lactose, chloride, sodium, and potassium in milk [15]. Pearson correlation coefficients demonstrated a highly significant negative relation between lactose content and freezing-point depression for all examined milk samples (r = −0.837 and *p* < 0.001). The average freezing-point depression (−0.515 °C), for both the production system and the season, was higher than the recorded values of the Hellenic Agricultural Organization-Demeter [25], which were −0.528 °C for the year 2019 and −0.527 °C for the year 2020 in relation to raw milk. Furthermore, Zagorska and Ciprovica [67] also observed a lower freezing-point depression in pasteurized whole-fat milk (−0.525 °C) compared to the results of the present study.

To summarize, the season significantly influenced milk pH, electrical conductivity, Brix value, and freezing-point depression, whereas the farming system affected only electrical conductivity. These differences can be linked to factors like animal diet, environmental temperature, and the presence of added water.

## 4. Conclusions

This is the first study to explore farming systems and seasonal effects on retail cow milk quality in Greece. Samples were categorized as conventional or organic based on product labels, and the focus was solely on Greek-produced milk.

In conclusion, the composition of the milk was predominantly unaffected by the farming system, with the exception of higher fat content in organically produced milk. However, when examining the seasonal aspect, a significant impact on ash, protein, and added-water content was evident. Neither the farming system nor the season exhibited a substantial effect on fatty acid composition, despite some variations in individual fatty acid levels, which did not result in changes in lipid classes or nutritional indices related to healthy-fat consumption, except for a lower AI level in organically produced milk. Regarding antioxidant activity, the production system favored conventionally produced milk, particularly in terms of free radical scavenging (DPPH), while the season significantly influenced both DPPH and ferric reducing-antioxidant power (FRAP). Furthermore, the physicochemical characteristics of the milk were more significantly affected by the season rather than the farming system. Season significantly influenced various aspects of retail milk, including its chemical composition, antioxidant profile, and physicochemical traits. In contrast, the production system primarily impacted the fatty acid profile, with a less-pronounced effect on other milk characteristics.

A comprehensive understanding of the effect of the farming system and season on retail milk, requires the examination of other factors, such as the content of micronutrients and the sensory characteristics of milk, i.e., flavour and taste. Parameters such as product variability within the same production batch and differences between branded and private-label products within the same farming system should also be studied.

## Figures and Tables

**Table 1:** Cow milk production (1000 kg) according to the farming system (years 2020–2023) (Hellenic Agricultural Organization-Demeter [14]).

Year	Farming System		Total
	Conventional	Organic	
2020	652,239	618 ^1^	652,857
2021	642,769	25,555	668,324
2022	618,276	24,795	643,071
2023 ^2^	417,435	15,177	432,612

^1^ Available data for the last trimester and for limited regional units; ^2^ available data until August.

**Table 2:** Characteristics of the collected milk samples.

Brand No	Production System	Shelf Life	Label
1	Conventional	7 days	Branded
2	Conventional	7 days	Branded
3	Conventional	7 days	Branded
4	Conventional	7 days	Private
5	Conventional	7 days	Private
6	Conventional	7 days	Branded
7	Conventional	7 days	Branded
8	Organic	10 days	Private
9	Organic	Extended shelf life (17 days)	Branded

**Table 3:** Declared chemical composition (g/100 mL) of retail cow milk purchased in Greece during the 12-month period from November 2019 to October 2020.

Brand No	Farming System	Fat	SFA ^1^	Protein	Carbohydrates	Sugars
1	Conventional	3.5	2.4	3.2	4.7	4.7
2	Conventional	3.5	2.3	3.2	4.6	4.6
3	Conventional	3.5	2.0	3.2	4.7	4.7
4	Conventional	3.5	2.4	3.2	4.7	4.7
5	Conventional	3.5	2.35	3.2	4.7	4.7
6	Conventional	3.9	2.6	3.4	4.7	4.7
7	Conventional	3.7	2.2	3.3	4.7	4.7
8	Organic	3.8	2.5	3.3	4.7	4.7
9	Organic	3.7	2.0	3.3	4.7	4.7
	Average	3.62	2.31	3.26	4.69	4.69

^1^ SFA, saturated fatty acids.

**Table 4:** Effect of production system and season on milk proximate composition.

Variable (g/100 g)	Farming System			Season					Significance	
	Conventional *n* = 84	Organic *n* = 24	SED_FS_	Spring *n* = 27	Summer *n* = 27	Autumn *n* = 27	Winter *n* = 27	SED_S_	FS ^1^	S ^2^
Moisture	87.57	87.43	0.079	87.61	87.57	87.42	87.39	0.112	NS	NS
Ash	0.68	0.68	0.007	0.69 ^b^	0.71 ^b^	0.69 ^b^	0.63 ^a^	0.009	NS	***
Fat	3.71	3.85	0.053	3.71	3.80	3.80	3.82	0.075	**	NS
Protein	3.39	3.38	0.023	3.36 ^b^	3.28 ^a^	3.37 ^b^	3.51 ^c^	0.032	NS	***
Lactose	4.66	4.66	0.019	4.62 ^a^	4.65 ^a^	4.71 ^b^	4.65 ^a^	0.028	NS	*
Total solids	12.43	12.57	0.079	12.39	12.43	12.58	12.61	0.112	NS	NS
Solids non-fat	8.72	8.72	0.039	8.68 ^ab^	8.64 ^a^	8.78 ^b^	8.78 ^b^	0.055	NS	*
Added water	2.06	1.94	0.365	2.75 ^b^	2.99 ^b^	1.01 ^a^	1.23 ^a^	0.517	NS	***

^1^ Farming system; ^2^ season; superscripts a, b, c differ at *p* < 0.05; * = *p* < 0.05; ** = *p* < 0.01; *** = *p* < 0.001; NS = non-significant; SED_FS_: standard error of difference between the two-farming systems; SED_S_: standard error of difference between the four seasons.

**Table 5:** Effect of production system and season on milk fatty acid composition (% of total identified fatty acids).

Variable	Farming System			Season					Significance	
	Conventional *n* = 84	Organic *n* = 24	SED_FS_	Spring *n* = 27	Summer *n* = 27	Autumn *n* = 27	Winter *n* = 27	SED_S_	FS ^1^	S ^2^
Fatty acid										
C4:0	1.29	1.40	0.080	1.27 ^a^	1.71 ^b^	1.16 ^a^	1.22 ^a^	0.113	NS	***
C6:0	1.25	1.13	0.136	1.24	1.13	1.01	1.20	0.193	NS	NS
C8:0	0.96	0.86	0.099	1.04	0.79	0.81	1.01	0.140	NS	NS
C10:0	2.33	2.13	0.139	2.58 ^c^	1.98 ^a^	1.99 ^ac^	2.36 ^bc^	0.197	NS	**
C11:0	0.39	0.24	0.122	0.22	0.33	0.43	0.28	0.174	NS	NS
C12:0	2.98	2.80	0.135	3.20 ^c^	2.60 ^a^	2.73 ^ac^	3.04 ^bc^	0.191	NS	**
C13:0	0.95	0.59	0.239	8.88	0.53	0.64	0.82	0.339	NS	NS
C14:0	10.74	9.92	0.316	10.79 ^b^	9.55 ^a^	10.47 ^b^	10.51 ^b^	0.447	*	*
C14:1	1.10	0.97	0.038	1.06	0.95	1.07	1.06	0.053	**	NS
C15:0	1.08	1.01	0.033	1.09 ^b^	0.96 ^a^	1.08 ^b^	1.06 ^b^	0.046	*	**
C15:1	0.71	0.33	0.702	0.30	0.31	0.37	0.28	0.992	NS	NS
C16:0	34.50	34.19	0.912	33.48	34.00	35.98	33.91	1.289	NS	NS
C16:1	1.33	1.20	0.096	1.56 ^b^	0.94 ^a^	0.95 ^a^	1.59 ^b^	0.135	NS	***
C17:0	0.62	0.61	0.035	0.61	0.62	0.67	0.58	0.049	NS	NS
C17:1	0.50	0.29	0.336	0.23	0.52	0.37	0.25	0.476	NS	NS
C18:0	10.35	11.72	0.429	11.01	11.75	10.51	10.88	0.607	**	NS
C18:1 *trans-11* (VA)	1.44	1.60	0.557	1.41	2.02	1.40	1.27	0.787	NS	NS
C18:1 *cis*-9	23.23	23.85	0.696	22.66	23.59	24.48	23.44	0.984	NS	NS
C18:2 *n-6 trans*	0.69	0.70	0.110	0.68	0.75	0.70	0.66	0.156	NS	NS
C18:2 *n-6 cis*	2.23	2.80	0.171	2.55	2.83	2.36	2.32	0.242	**	NS
C18:3 *n-6*	0.59	0.41	0.069	0.41	0.42	0.63	0.53	0.097	*	NS
C18:3 *n-3*	0.37	0.40	0.073	0.19 ^a^	0.33 ^ab^	0.55 ^b^	0.48 ^b^	0.102	NS	**
C18:2 *cis-9 trans-11* (CLA)	0.47	0.44	0.041	0.43	0.48	0.46	0.45	0.058	NS	NS
C20:0	0.32	0.22	0.056	0.24	0.20	0.28	0.33	0.078	NS	NS
Lipid class										
SCSFA ^3^	5.69	5.44	0.418	6.40 ^c^	5.18 ^ab^	4.60 ^a^	6.08 ^bc^	0.591	NS	*
MCSFA ^4^	50.21	48.45	1.114	49.64	47.59	50.77	49.33	1.574	NS	NS
LCSFA ^5^	11.14	12.49	0.437	11.83	12.43	11.26	11.75	0.618	**	NS
SFA ^6^	67.04	66.38	1.218	67.87	65.20	66.63	67.16	1.713	NS	NS
MUFA ^7^	29.08	29.08	1.119	27.97	30.35	29.44	28.55	1.583	NS	NS
PUFA ^8^	3.88	4.54	0.227	4.16	4.45	3.92	4.29	0.321	**	NS
UFA ^9^	32.96	33.62	1.214	32.13	34.80	33.37	32.84	1.717	NS	NS
OCFA ^10^	3.14	2.65	0.173	3.22 ^b^	2.62 ^a^	2.76 ^ab^	2.99 ^ab^	0.244	*	**
*n*-3	0.27	0.31	0.073	0.17 ^a^	0.26 ^ab^	0.30 ^ab^	0.43 ^b^	0.103	NS	NS
*n*-6	3.22	3.69	0.178	3.52 ^ab^	3.82 ^b^	3.02 ^a^	3.45 ^ab^	0.251	*	NS
Δ^9^—desaturase activity										
C14:1/C14:0	0.09	0.08	0.011	0.09	0.09	0.09	0.09	0.016	NS	NS
C16:1/C16:0	0.04	0.03	0.008	0.04	0.03	0.02	0.05	0.011	NS	NS
C18:1 *cis-9*/C18:0	0.68	0.64	0.007	0.66	0.67	0.64	0.68	0.010	NS	NS

^1^ Farming system; ^2^ season; ^3^ short-chained saturated fatty acids; ^4^ medium-chained saturated fatty acids; ^5^ long-chained saturated fatty acids; ^6^ saturated fatty acids; ^7^ monounsaturated fatty acids; ^8^ polyunsaturated fatty acids; ^9^ unsaturated fatty acids; ^10^ odd-chained fatty acids; superscripts a, b, c differ at *p* < 0.05; * = *p* < 0.05; ** = *p* < 0.01; *** = *p* < 0.001; NS = non-significant; SED_FS_: standard error of difference between the two-farming systems; SED_S_: standard error of difference between the four seasons.

**Table 6:** Effect of production system and season on milk fat nutritional indices.

Index	Farming System			Season					Significance	
	Conventional *n* = 84	Organic *n* = 24	SED_FS_	Spring *n* = 27	Summer *n* = 27	Autumn *n* = 27	Winter *n* = 27	SED_S_	FS ^1^	S ^2^
AI ^3^	2.50	2.23	0.090	2.48	2.26	2.26	2.46	0.127	*	NS
TI ^4^	3.30	3.07	0.105	3.32	3.20	3.02	3.21	0.148	NS	NS
h/H ^5^	0.59	0.59	0.073	0.56	0.68	0.53	0.59	0.104	NS	NS
HPI ^6^	0.39	0.43	0.013	0.40	0.43	0.38	0.42	0.018	NS	NS
PUFA/SFA ^7^	0.06	0.07	0.008	0.06	0.07	0.05	0.06	0.011	NS	NS

^1^ Farming system; ^2^ season; ^3.^ atherogenicity index; ^4^ thrombogenicity index; ^5^ hypocholesterolaemic: hypercholesterolaemic ratio; ^6^ heath-promoting index; ^7^ polyunsaturated fatty acid/saturated fatty acid ratio; * = *p* < 0.05; NS = non-significant; SED_FS_: standard error of difference between the two-farming systems; SED_S_: standard error of difference between the four seasons.

**Table 7:** Effect of production system and season on milk antioxidant profile.

Index	Farming System			Season					Significance	
	Conventional *n* = 84	Organic *n* = 24	SED_FS_	Spring *n* = 27	Summer *n* = 27	Autumn *n* = 27	Winter *n* = 27	SED_S_	FS ^1^	S ^2^
TPC (mg GAE/mL) ^3^	0.64	0.68	0.025	0.70 ^b^	0.65 ^ab^	0.63 ^a^	0.64 ^ab^	0.025	NS	NS
DPPH (μM TE/mL) ^4^	14.54	12.30	0.828	15.58 ^a^	13.27 ^ab^	12.51 ^b^	12.29 ^a^	0.828	**	*
FRAP (μM TE/mL) ^5^	26.09	24.87	1.547	27.94 ^b^	27.86 ^b^	21.81 ^a^	24.31 ^ab^	1.547	NS	*
ABTS (μM TE/mL) ^6^	541.94	545.27	17.064	519.86	528.99	580.55	545.01	17.064	NS	NS

^1^ Farming system; ^2^ season; ^3^ total phenolic content expressed in mg of gallic acid equivalents (GAE)/mL; ^4^ 2,2-Diphenyl-1-picrylhydrazyl radical scavenging activity expressed as μΜ of Trolox equivalents (TE)/mL; ^5^ ferric reducing-antioxidant power expressed as μΜ of Trolox equivalents (TE)/mL; ^6^ 2-Azino-bis-3-ethylbenzothiazoline-6-sulfonic acid radical scavenging activity expressed as μΜ of Trolox equivalents (TE)/mL; superscripts a, b differ at *p* < 0.05; * = *p* < 0.05; ** = *p* < 0.01; NS = non-significant; SED_FS_: standard error of difference between the two farming systems; SED_S_: standard error of difference between the four seasons.

**Table 8:** Effect of production system and season on milk physicochemical characteristics.

Variable	Farming System			Season					Significance	
	Conventional *n* = 84	Organic *n* = 24	SED_FS_	Spring *n* = 27	Summer *n* = 27	Autumn *n* = 27	Winter *n* = 27	SED_S_	FS ^1^	S ^2^
pH	6.74	6.74	0.009	6.78^b^	6.71 ^a^	6.74 ^a^	6.73 ^a^	0.013	NS	***
Electrical Conductivity (mS/cm)	4.85	4.77	0.022	4.71 ^a^	4.87 ^c^	4.88 ^cd^	4.78 ^b^	0.032	**	***
Refractive index	1.351	1.351	0.001	1.350 ^a^	1.350 ^ab^	1.351 ^b^	1.352 ^c^	0.001	NS	***
Brix (°Bx)	12.008	11.96	0.119	12.25 ^b^	11.57 ^a^	11.85 ^a^	12.27 ^b^	0.169	NS	***
FPD(°C) ^3^	−0.515	−0.516	0.002	−0.511 ^b^	−0.509 ^b^	−0.521 ^a^	−0.520 ^a^	0.003	NS	***

^1^ Farming system; ^2^ season; ^3^ freezing-point depression; superscripts a, b, c, d differ at *p* < 0.05; ** = *p* < 0.01; *** = *p* < 0.001; NS = non-significant; SED_FS_: standard error of difference between the two-farming systems; SED_S_: standard error of difference between the four seasons.

## Data Availability

The data presented in this study are contained within the article.

## Abbreviations

AI	Atherogenicity index
CLA	Conjugated linoleic acid
h/H	Hypocholesterolemic/hypercholesterolemic ratio
HPI	Health-promoting index
LA/ALA	Linoleic acid/α-linolenic acid ratio
LCSFA	Long-chained saturated fatty acids
MCSFA	Medium-chained saturated fatty acids
MUFA	Monounsaturated fatty acids
OCFA	Odd-chain fatty acids
PUFA	Polyunsaturated fatty acids
SCSFA	Short-chained saturated fatty acids
SFA	Saturated fatty acids
TI	Thrombogenicity index
VA	Vaccenic acid
